# Treatment sequence of stem cell delivered heterologous oncolytic virus impact on tumor microenvironment in immunocompetent ovarian cancer model

**DOI:** 10.3389/fimmu.2026.1898617

**Published:** 2026-08-31

**Authors:** Eslam E. Abd El-Fattah, Linda Flores, Jacqueline Lara, Gary Ngai, Rachael Mooney, Karen Aboody

**Affiliations:** Department of Stem Cell Biology and Regenerative Medicine, Beckman Research Institute of City of Hope, Duarte, CA, United States

**Keywords:** adenovirus, immune modulation, oncolytic virotherapy, ovarian cancer, vaccinia virus

## Abstract

**Introduction:**

Ovarian cancer (OC) is a biologically heterogeneous malignancy associated with poor clinical outcomes. Oncolytic viruses (OVs) have emerged as a promising immunotherapeutic strategy; however, optimal approaches to maximize their antitumor efficacy—especially in combinatorial or sequential settings—remain insufficiently defined. To determine how the sequence of administration of two clinically relevant, stem cell–delivered OV platforms influences tumor control and immune remodeling in an immunocompetent OC model.

**Methods:**

We investigated the sequence of two clinically relevant products: neural stem cell delivered conditionally replication competent adenovirus (NSC.CRAd-S-pk7; NNV24) and a mesenchymal stem cell delivered vaccinia virus (MSC.VP001; SNV1). Female C57BL/6 mice were randomized into three groups: untreated tumor-bearing controls, NNV24 followed by SNV1 (NNV24+SNV1), or SNV1 followed by NNV24 (SNV1+NNV24). Therapeutic efficacy and immune responses were assessed by overall survival, histopathological evaluation, flow cytometric analysis of dendritic cell (DC) subsets and cytotoxic T lymphocytes (CTLs), and bulk RNA sequencing.

**Results:**

Treatment regimens initiated with NNV24 significantly prolonged survival compared with SNV1-first sequences. NNV24+SNV1 treatment resulted in enhanced intratumoral viral localization, increased infiltration of CTLs, mature DCs, and concomitant with a reduction in tolerogenic DC populations. Transcriptomic profiling corroborated these findings through revealing suppression of immunoregulatory and tolerogenic pathways signaling in comparison to SNV1+NNV24 group.

**Discussion:**

The treatment sequence of heterologous OV has a significant effect on the tumor microenvironment. NNV24+SNV1 promotes robust antitumor immunity and improved survival, whereas the reverse sequence compromises immune activation and therapeutic response. These findings establish a mechanistic framework for rational sequencing of OV-based immunotherapies.

## Graphical abstract

**Figure:**
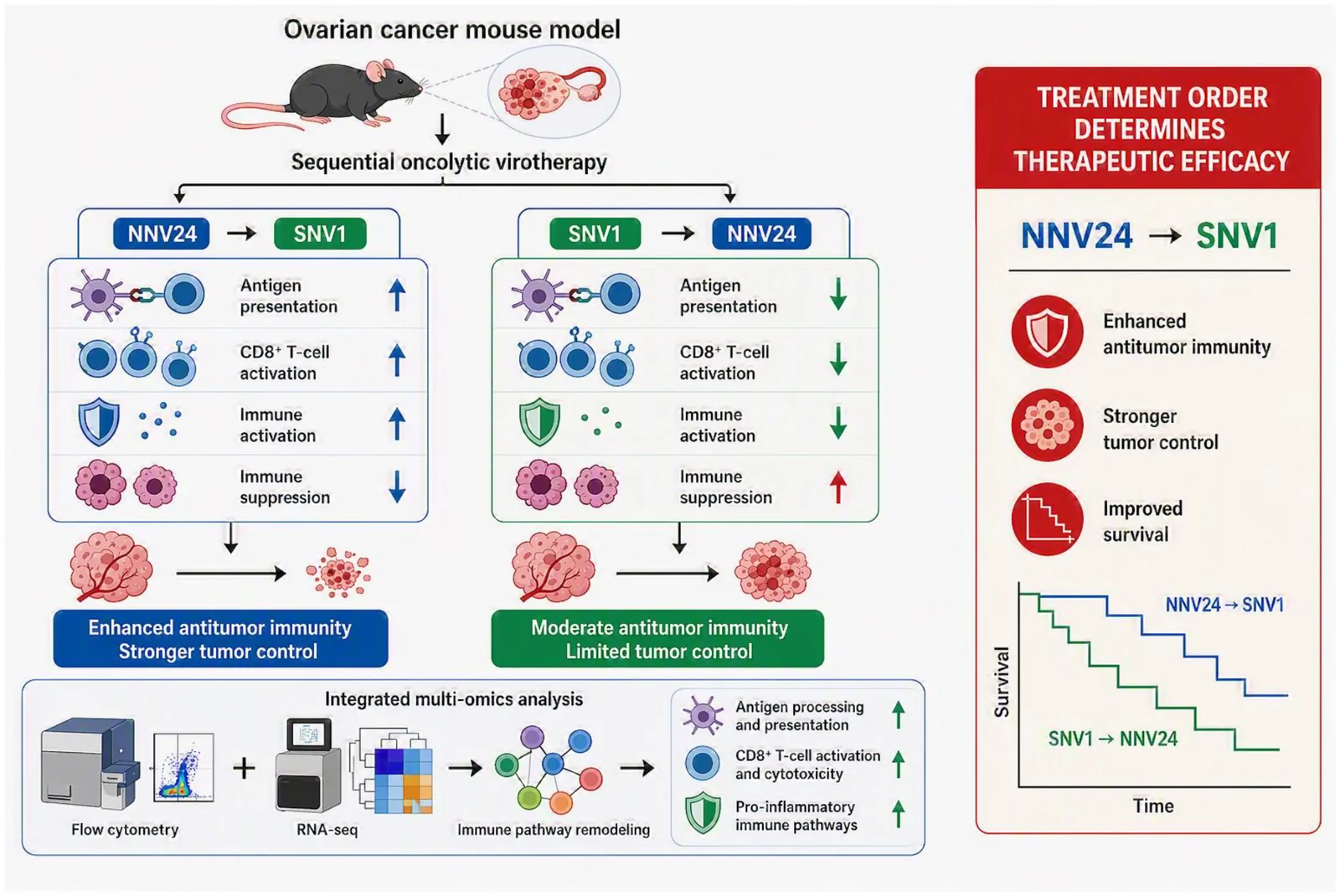
Treatment sequence shapes anti-tumor immunity during sequential oncolytic virotherapy. In an orthotopic ovarian cancer model, NNV24→SNV1 induced superior immune priming characterized by enhanced antigen presentation, increased CD8^+^ T-cell activation, reduced immune suppression, and stronger tumor control compared with SNV1→NNV24. Multi-omics analyses identified immune pathway remodeling associated with improved antitumor immunity and survival, highlighting treatment order as a key determinant of therapeutic efficacy.

## 1 Introduction

Ovarian cancer (OC) is a biologically heterogeneous malignancy that disproportionately affects postmenopausal women and remains a leading cause of gynecologic cancer related mortality worldwide (1). Most patients are diagnosed at advanced stages with widespread intraperitoneal (i.p.) dissemination, largely due to the absence of effective early detection strategies. Consequently, long-term survival remains poor despite advances in cytoreductive surgery, and chemotherapeutic approaches (2). Global epidemiologic trends emphasize the persistent disease burden and underscore the urgent need for novel therapeutic approaches capable of inducing durable clinical responses across OC histologic subtypes, particularly high-grade serous OC, which accounts for the majority of disease related deaths (3).

Oncolytic viruses (OVs) represent a promising therapeutic modality by integrating direct tumor selective lysis with the induction of antitumor immunity. Through the release of tumor associated antigens and proinflammatory signals, OVs can convert immunologically “cold” tumors into inflamed, antigen rich tumor microenvironment (TME) that facilitate dendritic cell (DC) activation, cytotoxic T cell infiltration, and enhanced responsiveness to immune checkpoint blockade (4). Nevertheless, clinical translation of OV monotherapy has been constrained by barriers that include inefficient intratumoral delivery, rapid host antiviral immune clearance, and tumor intrinsic resistance mechanisms. These limitations highlight the necessity for optimized delivery platforms and rational administration strategies to fully harness the therapeutic potential of OVs (5).

Adenovirus and vaccinia virus represent particularly promising OV backbones for the treatment of OC owing to their complementary biological and translational properties. Conditionally replicating adenoviruses (CRAds) can be genetically engineered for tumor restricted replication and transgene expression, enabling localized tumor lysis while enhancing antigen release and presentation within TME (6). Vaccinia virus, by contrast, possesses a large genomic payload capacity, supports rapid and robust intratumoral replication, and is amenable to both regional and systemic administration, facilitating widespread engagement of disseminated peritoneal tumor foci. Recent advances in vector engineering, including arming strategies and immunomodulatory transgenes, have further amplified the immunostimulatory potential of both adenoviral and vaccinia based OV platforms (7).

Stem cell-based delivery strategies address several key obstacles to OV translation, particularly host immune neutralization and inefficient viral dissemination across multifocal i.p. disease. Stem cell-based delivery exhibits intrinsic tumor homing capacity and function as intratumoral “virus factories, “ enabling sustained viral amplification while circumventing systemic barriers such as liver sequestration and antibody mediated clearance. Tumor-tropic neural stem cells (NSCs) can shield adenoviral vectors from premature immune clearance, allow for viral amplification, promote targeted intratumoral homing, and facilitate the handoff of replication competent viral payloads within tumor sites (8, 9).

Notably, NSC delivered CRAd-S-pk7 has demonstrated feasibility, safety, and immunologic activity in first in-human clinical studies for glioma, as well as enhanced i.p. delivery and antitumor efficacy in preclinical models of ovarian metastasis (10, 11). In OC, mesenchymal stem cell (MSC)-mediated OV delivery has been shown to intensify i.p. targeting and amplify local oncolysis and immune remodeling. Collectively, preclinical investigations and translational reviews support MSC-based carrier platforms as an effective strategy for vaccinia and other OVs to enhance the therapeutic index and antitumor immune activation (12–15).

A major and persistent limitation of OV-based therapy is the challenge of effective redosing. Repeated administration of the same viral platform rapidly elicits neutralizing antibodies and activates innate antiviral defense programs, including type I interferon signaling, which collectively diminish viral persistence, intratumoral replication, and therapeutic payload delivery upon subsequent dosing (16, 17). Heterologous OV sequencing—defined by the sequential administration of antigenically distinct viral platforms—offers a rational strategy to overcome these barriers. Analogous to heterologous prime–boost vaccination paradigms, this approach can evade vector specific immunity while broadening and sustaining tumor specific T-cell- responses (18). Consistent with this rationale, preclinical studies have demonstrated that sequential delivery of distinct OVs enhances antitumor efficacy, immune infiltration, and functional immune activation relative to monotherapy or homologous viral redosing, underscoring the importance of sequence dependent optimization in OV-based- immunotherapy (19).

Given the predominant i.p. dissemination and frequent omental involvement characteristic of OC, the ID8 syngeneic, immunocompetent mouse model represents a clinically relevant platform for investigating OV delivery, regional tumor biology, and antitumor immune responses in the context of intact host immunity (20). ID8 tumors recapitulate key pathological and immunologic features of human OC, including ascites formation, macrophage dominated immunosuppressive niches, and widespread peritoneal tumor implants. These attributes enable mechanistic interrogation of TME remodeling, viral distribution, and effector immune cell activation following i.p. therapeutic intervention, providing a robust framework for preclinical evaluation of dominated immunosuppressive niches, and widespread peritoneal tumor metastasis (21).

Here, we evaluated the impact of treatment sequence in the sequential administration of two clinically relevant, stem cell–OV delivery platforms: NSC delivered CRAd-S-pk7 adenovirus (NNV24) and MSC delivered VP001 vaccinia virus (SNV1). Female C57BL/6 mice bearing i.p. ID8.ffluc OC tumors were treated with either NNV24 followed by SNV1 (NNV24 + SNV1) or the reverse sequence (SNV1 + NNV24), administered at two-week intervals to approximate clinically feasible redosing paradigms. Therapeutic efficacy and immune modulation were comprehensively assessed using overall survival and longitudinal bioluminescence imaging (BLI), alongside histopathologic and immunohistochemical analyses to evaluate viral localization, tumor cell proliferation, apoptosis, and macrophage polarization. Immune profiling was further performed by flow cytometric quantification of DC maturation states and cytotoxic CD8^+^ T cell responses, complemented by bulk RNA sequencing to capture pathway level transcriptional reprogramming within the TME.

We hypothesized that initiating heterologous therapy with an adenoviral OV would maximize early intratumoral antigen exposure and activate antigen processing and presentation pathways, thereby priming DCs and establishing a permissive inflammatory baseline within the TME. In this context, subsequent administration of vaccinia virus (SNV1) was expected to function as an immunologic “boost, “ enhancing spatially distributed oncolysis, sustaining proinflammatory signaling, and reinforcing cytotoxic T lymphocyte (CTL) expansion and infiltration while concurrently suppressing tolerogenic immune compartments.

By strategically integrating clinically relevant stem cell carriers—NSCs for adenoviral delivery and MSCs for vaccinia delivery—within a heterologous OV sequencing framework, this study seeks to define a mechanistically grounded and sequence optimized oncolytic immunotherapy paradigm for OC. These findings aim to provide actionable insights to inform the rational design and clinical translation of next generation OV based immunotherapeutic strategies.

## 2 Materials and methods

### 2.1 Cell culture

The v-*myc* immortalized, tumor-tropic, clonal human HB1.F3.CD21 NSC line (with demonstrated safety in human glioma trials) was obtained from Dr. Seung Kim (University of British Columbia, Canada). The NSCs were further modified to produce NSC.eGFP.ffluc cells expressing green fluorescent protein (eGFP) and firefly luciferase (ffluc), as previously described (22, 23).

The ID8 murine ovarian cancer line was obtained from Dr. Katherine Roby (University of Kansas). These cells were further modified using a PJ01668-eGFP-ffluc-epHIV7 lentiviral vector (159e6 TU/ML; VF0716) generously provided by Dr. Christine Brown (City of Hope) to produce ID8.eGFP.ffluc cells. The NSC and ID8 cell lines were cultured in DMEM (Invitrogen) supplemented with 10% fetal bovine serum (Gemini Bio), 1% L-glutamine (Invitrogen), and 1% penicillin-streptomycin (Invitrogen). All cells were maintained at 37 °C in a humidified incubator (Thermo Electron Corporation) containing 6% CO2 and passaged using a 0.25% trypsin and EDTA solution (Invitrogen) when they reached 80% confluency, and media were changed every 2–3 days. SNV-1 was “provided as a kind gift from Calidi Biosciences” (23).

### 2.2 Study design and animal model

Twenty-four female C57BL/6 mice (6–8 weeks old, 20–25 gm each, from Charles River/NCI) were used to establish an immunocompetent ovarian cancer model. Animals were housed under pathogen free housing and allowed free access to food and water. This animal study was conducted under an approved protocol by the Institutional of Animal Care and Use Committee (City of Hope/Beckman Research Institute, Duarte, CA).

Mice were injected i.p. with 5×10^6^ ID8.ffluc cells (firefly luciferase-expressing ID8 murine OC cells) to enable longitudinal bioluminescence imaging (BLI) of tumor burden. Mice that did not show BLI signal were excluded from the study. Animals were randomized into three treatment groups (8 per group):

1. Tumor only control
2. NNV24+SNV1 (NNV24 followed by SNV1)
3. SNV1+NNV24 (SNV1 followed by NNV24)

### 2.3 Oncolytic virus formulations and delivery

NNV24 consisted of NSCs loaded with CRAd-S-pk7 adenovirus (24), and SNV1 consisted of MSCs loaded with VP001 vaccinia virus. Each OV dose contained 2×10^6^ carrier cells and was administered i.p. at two-week intervals. NNV24 + SNV1: NNV24 on day 7, SNV1 on day 21. SNV1+NNV24: SNV1 on day 7, NNV24 on day 21. We choose the doses based on preliminary optimization experiments.

Mice were monitored daily for clinical signs and body weight to assess tolerability. Survival was monitored until humane endpoints were reached, and Kaplan–Meier curves were generated for each group. At the end of the study, mice were euthanized in accordance with the 2020 AVMA Guidelines for the Euthanasia of Animals. Mice were euthanized via carbon dioxide inhalation at a displacement rate of 30 to 70% chamber volume/min. After cessation of respiratory movement, a bilateral thoracotomy was performed to collect up to 0.5 ml of blood via cardiocentesis. Blood and omentum (primary site of OC dissemination) were collected. Omental tissue was divided into pathology, flow cytometry and RNA sequencing.

### 2.4 Live-animal imaging

Tumor burden was evaluated via BLI. Using Lago X (Spectral Instruments Imaging, Tuson, AZ), the maximum signal from a region of interest was measured using Aura 5 software (Spectral Instruments Imaging). For all *in vivo* imaging, mice were given a 150ul injection of D-Luciferin potassium salt (28.57mg/ml, GoldBio, St Louis, MO). After 7 min the mice were anesthetized in an induction chamber with 2% to 4% isoflurane (Pivetal Isoflurane, Patterson Veterinary, Loveland, CO) in oxygen at 1.5 L/min and then transferred to an imaging box and maintained under isoflurane anesthesia throughout the imaging process.

### 2.5 Hematoxylin–eosin staining

Flash frozen-omental tissue was processed by standard histology. Briefly, tissues were fixed in 4% paraformaldehyde for 48 h, then preserved in 30% sucrose and embedded in OCT. Sections (10 μm) were cut, mounted on charged slides. Nuclei were stained with hematoxylin, differentiated and “blued” in an alkaline solution, then counterstained with eosin Y. Slides were dehydrated in ascending ethanol, cleared in xylene, and cover slipped with a resinous medium. 3–4 samples were used for analysis of each group.

### 2.6 Immunohistochemistry and immunofluorescence examination

Flash frozen omental slides prepared according to 2.4. were stained with antibodies for Ki-67 (MA5-14520, Invitrogen, USA), CD68 (PA5-78996, Invitrogen, USA), TNF-α (NBP1-19532, Novus Biologicals, USA), adenoviral Hexon protein (AB1056, Sigma Millipore, USA), and vaccinia virus markers (ab35219, Abcam, USA). Cleaved caspase-3 (9661S, Cell Signaling, USA) immunofluorescence was performed to assess apoptosis. 3–4 samples were used for analysis of each group.

### 2.7 Immunophenotyping via flow cytometry

Single-cell suspensions were prepared for immune profiling. Cells were stained with fluorochrome-conjugated antibodies against CD3 (Monoclonal Antibody (17A2), APC, eBioscience™, Thermofisher, USA), CD8 (Brilliant Violet 421™ anti-mouse CD8a Antibody, 100738, Biolegend, USA), CD11c (Monoclonal Antibody (N418), PE-Cyanine7, eBioscience™, ThermoFisher, USA), and CD83 (Monoclonal Antibody (Michel17), eFluor™ 660, eBioscience™, ThermoFisher, USA). Data were acquired on a BD LSRFortessa and analyzed using BD FACSDiva™ Software. 5–6 samples were used for analysis of each group.

### 2.8 RNA sequencing

We analyzed 9 tumor samples from the three groups, three per each group. Total RNA was extracted from omentum cell pellets using the RNeasy Plus Mini Kit (Qiagen, USA). The quality of the obtained total RNA was evaluated by calculating the DV200 index, measured with the RNA 6000 Pico kit (Agilent Technologies). RNA-seq libraries were constructed through Epicentre Ribo-zero™ rRNA Removal Kit (Epicentre Technologies, USA) to remove ribosomal RNA (rRNA) followed by reverse transcription to generate strand-specific complementary DNA libraries. Subsequently, paired-end sequencing (150 bp) was performed using Illumina NovaSeq 6000 S4, resulting in an average of 150 million total reads per library. RNA-seq data were aligned to the Mouse transcriptome using STAR-2.7.3a (25). Normalization of raw transcript counts and differential expression analysis were performed with DESeq2 1.42.0 (26) and the resulting *P* values were corrected for multiple testing using the Benjamini–Hochberg procedure. Normalized gene expression counts were analyzed with the fgsea48 (version 1.20.0) (26). Three samples were used for analysis of each group.

### 2.9 Statistical analysis

Statistical analysis was conducted using GraphPad Prism software version 9 (GraphPad Software Inc., La Jolla, CA, USA) and the data is presented as the mean ± standard deviation (SD). Differences between groups were analyzed by a one-way analysis of variance followed by Tukey’s Kramer *post hoc* multiple comparisons test. The Mantel-Cox test was used to establish the significance between-group differences in the Kaplan–Meier survival analysis. P values < 0.05 were considered statistically significant.

## 3 Results

### 3.1 NNV24 first sequencing improves survival and tumor control

**Figure 1:**
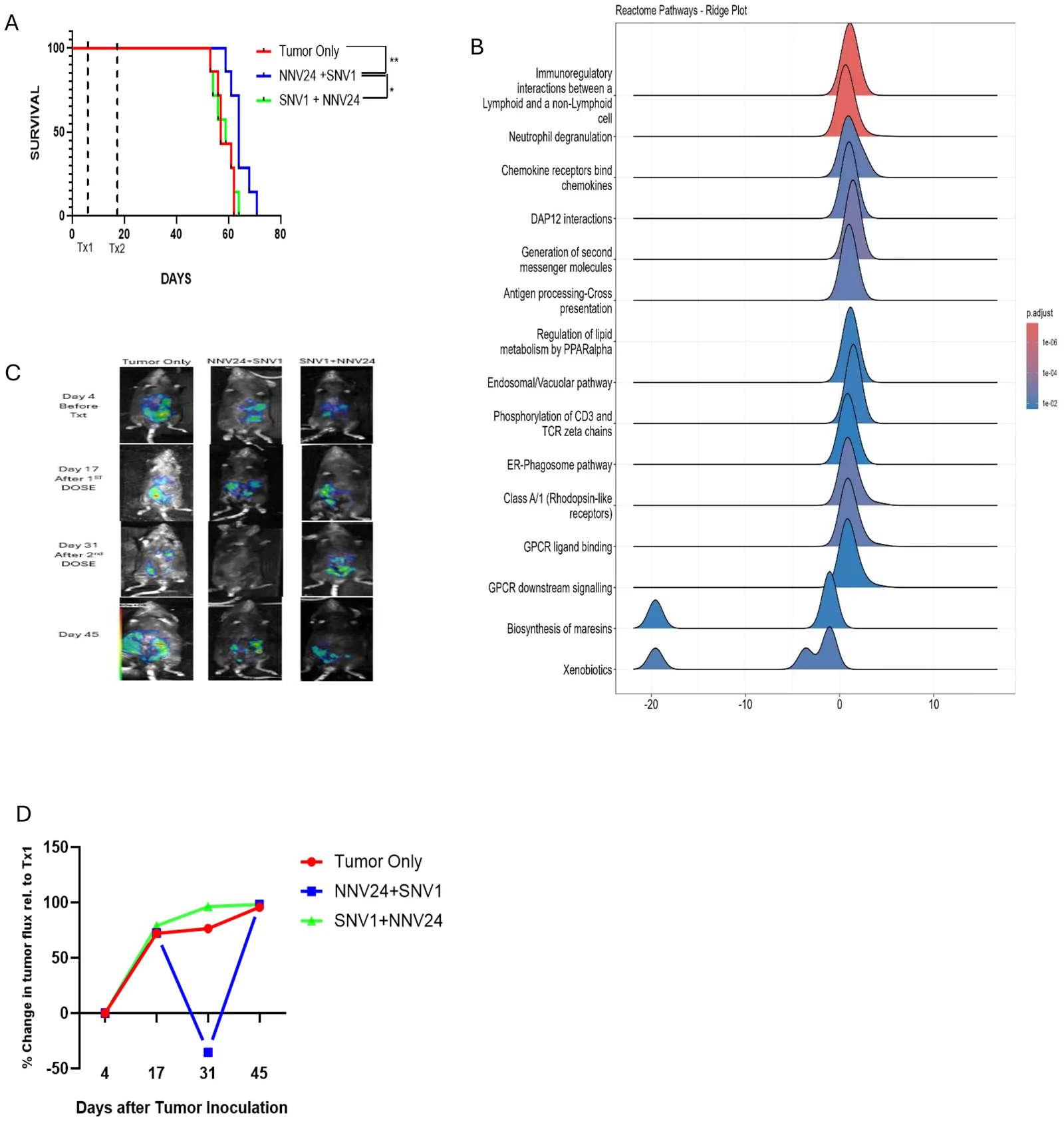
Therapeutic efficacy and pathway analysis. **(A)** Kaplan–Meier survival curves for C57BL/6 mice bearing i.p. tumors treated under four conditions: Tumor only, NNV24 + SNV1, and SNV1 + NNV24. Survival probability was monitored over time, with treatment doses indicated (Tx1–Tx2). Statistical significance between groups is shown (*p < 0.05; **p < 0.01). **(B)** Ridge plot of enriched Reactome pathways derived from RNA-seq analysis of tumor tissues, highlighting immune-related and signaling pathways significantly modulated by treatment. **(C)** Representative in vivo BLI of mice before treatment and after each dose (1st–4th), comparing tumor progression and response across groups. **(D)** Quantification of tumor BLI signal intensity (Region of Interest, ROI) across treatment groups, demonstrating relative tumor burden.

**Figure 2:**
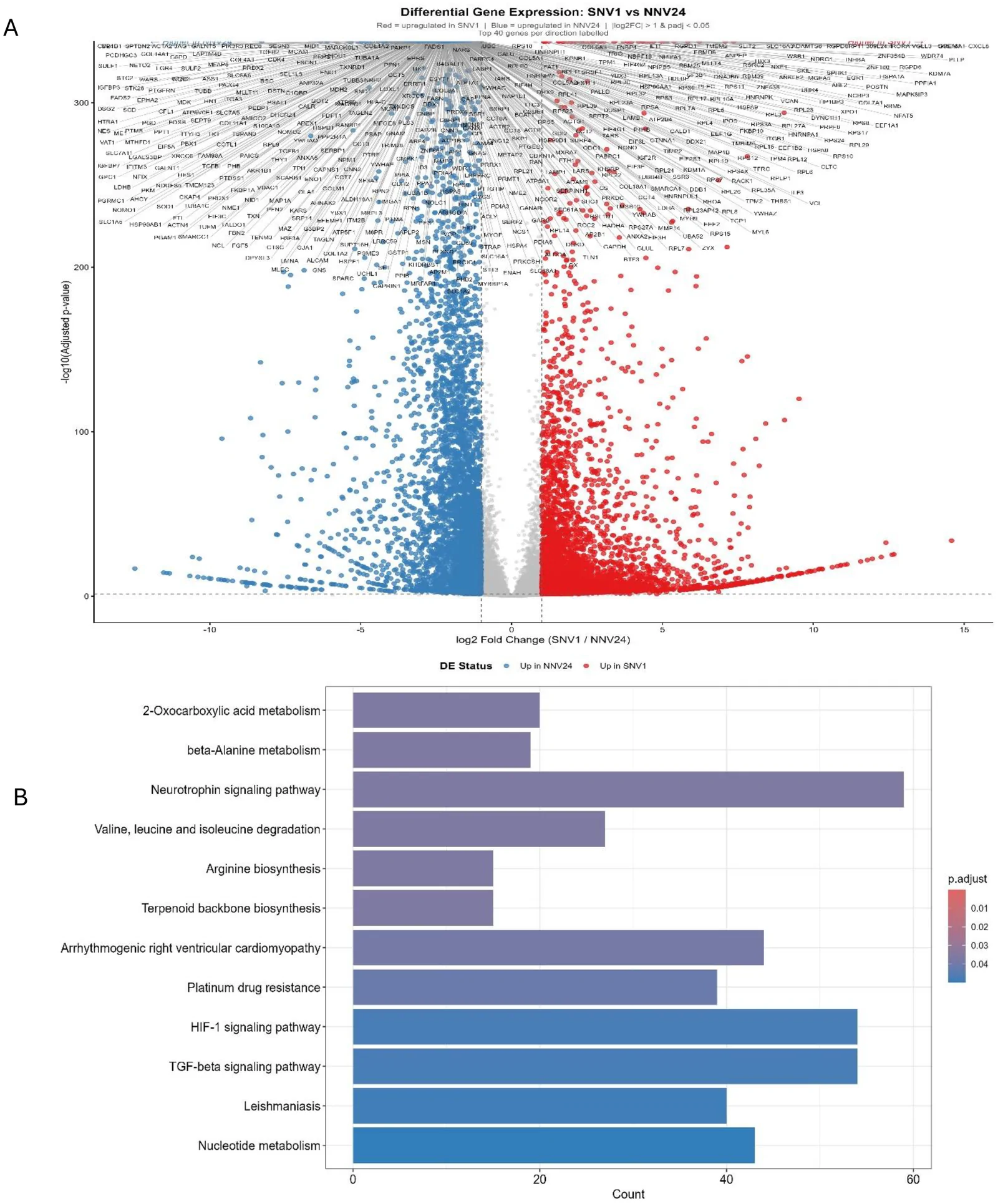
Transcriptomic and pathway-level differences between SNV1 and NNV24. **(A)** Volcano plot of differential gene expression between SNV1 and NNV24. Red and blue points indicate genes significantly upregulated in SNV1 and NNV24, respectively (|log2FC| > 1, padj < 0.05). Grey points represent non-significant genes. The top 40 differentially expressed genes per direction are labeled. Vertical dashed lines indicate the log2FC ± 1 threshold; horizontal dashed line indicates the padj = 0.05 cutoff. **(B)** KEGG pathway enrichment analysis of differentially expressed genes. Bar length represents the number of genes enriched per pathway (Count). Bar color reflects the adjusted p-value (padj), ranging from red (padj ≈ 0.01, most significant) to blue (padj ≈ 0.04, least significant). Only statistically significant pathways (padj < 0.05) are shown.

Kaplan–Meier analysis revealed that mice receiving NNV24 followed by SNV1 exhibited the longest survival, with the NNV24+SNV1 regimen providing a significant improvement in survival rate compared to SNV1+NNV24 (**Figure 1A**) while body weight remained stable across conditions (**Figure 1B**). Longitudinal BLI confirmed initial sharp tumor regression, with NNV24 + SNV1 regimens showing the most pronounced reduction in tumor signal which suggest high oncolytic activity in NNV24 + SNV1 prime boosting regimen in comparison to SNV1+NNV24 regimen which may be interpreted by better immune activation (**Figures 1C, D**). Transcriptomic pathway analysis and reactome pathway analysis demonstrated that changes in TME such as chemokine signaling, immunoregulatory interaction between lymphoid and non-lymphoid cells, and regulation of T-cell signaling are different across groups which may explain the difference in effect (**Figure 2E**). Collectively, these findings establish that initiating therapy with adenovirus followed by vaccinia provides more durable antitumor activity without systemic toxicity.

### 3.2 SNV1 exhibits different immune transcriptional profile relative to NNV24

Differential gene expression analysis between *in vitro* cultured SNV1 and NNV24 revealed significant immunological reprogramming (**Figure 2A**). Key upregulated genes in SNV1 included CXCL8, a neutrophil-recruiting chemokine, and TGFB2, implicating active remodeling of the immune TME. KEGG pathway enrichment analysis identified the HIF-1 and TGF-beta signaling pathways as the most significantly enriched (**Figure 2B**), both well-established drivers of immune evasion, cytotoxic T cell suppression, and regulatory T cell promotion. Together, these results suggest that SNV1 exhibits a transcriptional program dominated by immunosuppressive signaling, highlighting HIF-1 and TGF-β as potential targets for restoring antitumor immunity. This raised the question about the benefit of heterologous combination and the effect of different OV orders on cancer growth.

### 3.3 Enhanced oncolysis and viral distribution

**Figure 3:**
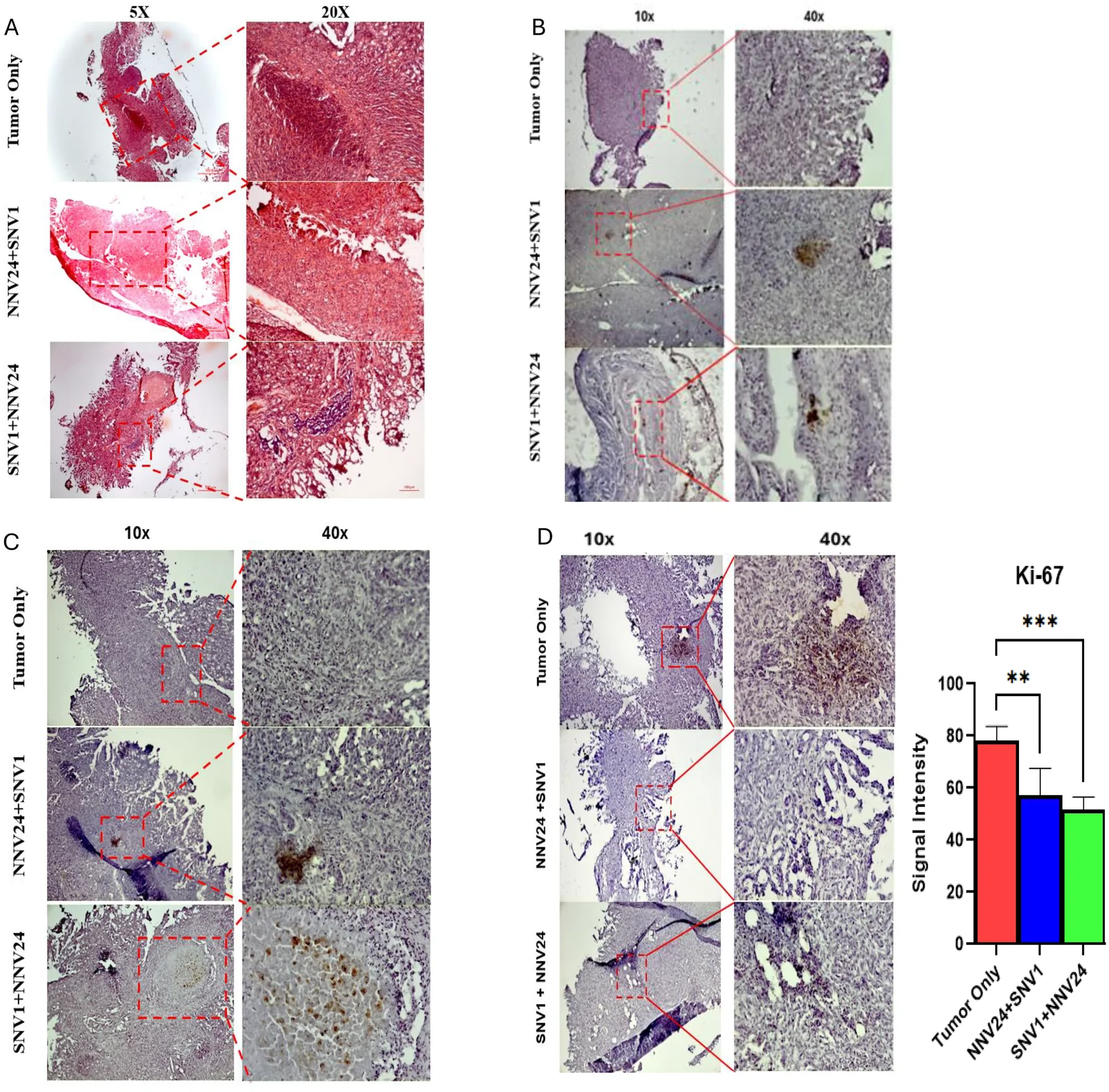
Histopathological and IHC Analysis of Tumor Tissues. **(A)** H&E staining of tumor sections from treatment groups: SNV1+NNV24, NNV24+SNV1, and tumor only. Representative images show overall tissue architecture and cellular morphology at low and high magnification. **(B)** IHC detection of Adenovirus in tumor sections. Similar magnifications (10× and 40×) highlight adenoviral presence and distribution following treatment. **(C)** IHC detection of vaccinia virus in tumor sections. Images at 10× and 40× magnification illustrate viral localization within tumor tissue across treatment groups. **(D)** Ki-67 staining to assess tumor cell proliferation. Representative images from each treatment group are shown with signal intensity bar chart illustrated (**p < 0.01, ***p < 0.001).

Histopathology corroborated these survival benefits. H&E staining revealed extensive tissue disruption and necrosis in NNV24-first tumors, whereas SNV1-first tumors retained dense architecture (**Figure 3A**). IHC confirmed robust intratumoral localization of both adenovirus and vaccinia virus in treated mice and first virus administration decreased the other, (**Figures 3B, C**). Ki-67 staining showed significantly reduced proliferation, with the two-dose NNV24+SNV1 schedule achieving the lowest Ki-67 signal compared with SNV1+NNV24, indicating superior suppression of tumor growth (**Figure 3D**).

### 3.4 Immune landscape remodeling

**Figure 4:**
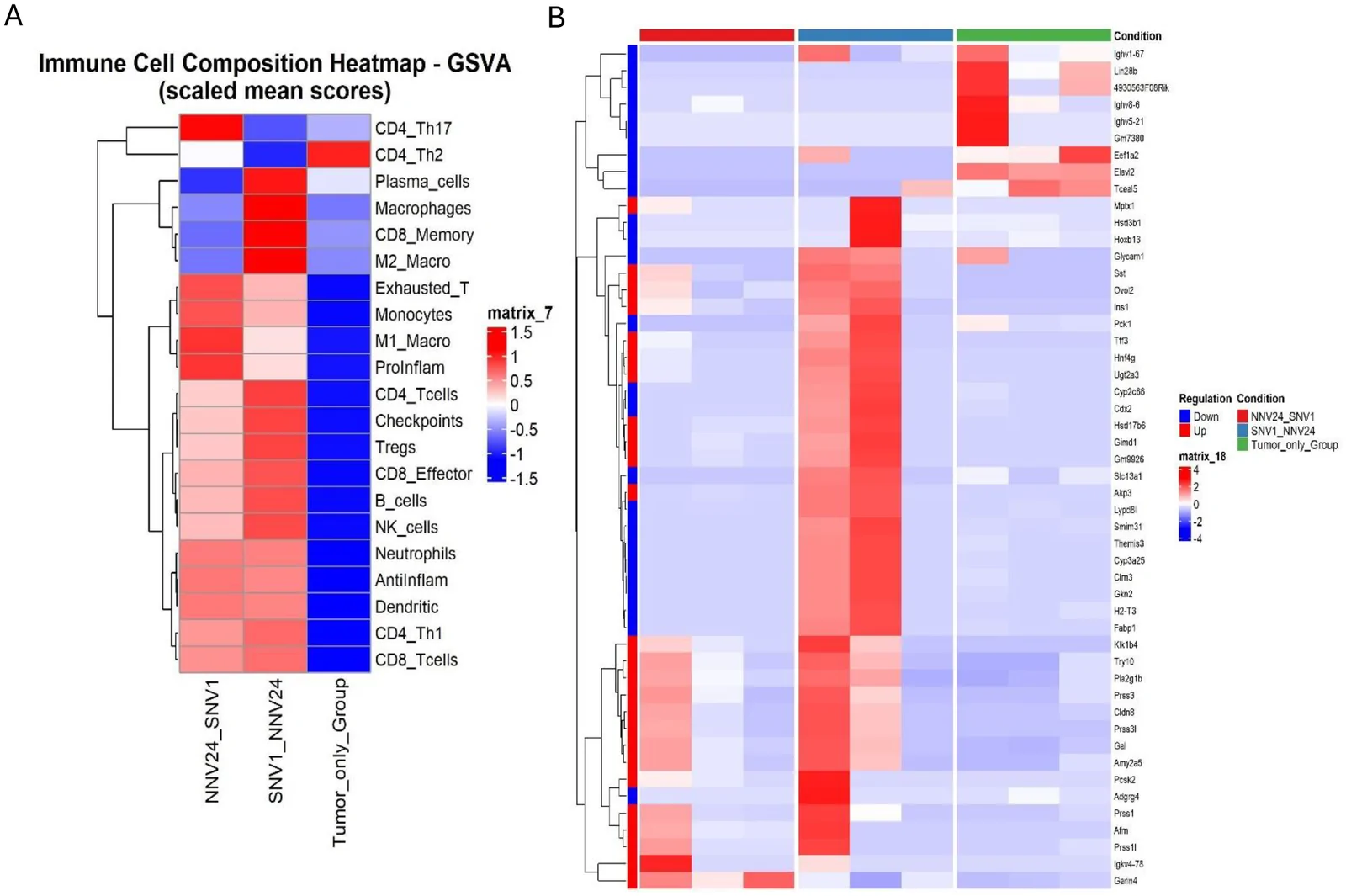
Immune cell composition and gene expression profiles across treatment conditions. **(A)** GSVA-based immune cell composition heatmap showing scaled mean enrichment scores across NNV24 + SNV1, SNV1 + NNV24, and tumor only groups. Red indicates higher enrichment and blue indicates lower enrichment. Immune cell populations are hierarchically clustered on the y-axis. **(B)** Heatmap of top differentially expressed genes across the three conditions. Color scale represents log2 expression values ranging from −4 (blue, downregulated) to +4 (red, upregulated). Genes and samples are hierarchically clustered. Condition annotations are indicated at the top.

GSVA-based immune profiling revealed that treatment order critically shapes the immune TME (**Figure 4A**). NNV24+SNV1 showed elevated enrichment of CD4_Th2, M2 macrophages, exhausted T cells, Tregs, and checkpoints, indicating a predominantly immunosuppressive and exhaustion prone state. In contrast, the tumor-only group displayed the lowest enrichment for cytotoxic populations including CD8 Effector T cells, NK cells, and M1 macrophages, consistent with an immune-cold phenotype. The SNV1+NNV24 group exhibited broadly reduced immune scores, suggesting active immune contraction post-treatment. Transcriptomic analysis corroborated these findings, with the tumor-only group showing upregulation of immunoglobulin-associated genes (*Ighv1-67*, *Ighv6-6*) reflecting baseline B cell activity, while NNV24+SNV1 displayed a broadly activated expression profile (**Figure 4B**). These results indicate that administering NNV24 before SNV1 generates a more immunologically active but exhaustion-prone TME, whereas the reverse order attenuates immune engagement.

To investigate how treatment sequence shapes the immune TME, we profiled immune marker expression across all samples and assessed intercellular immune coordination using GSVA-based Spearman correlation analysis across all three pairwise comparisons.

**Figure 5:**
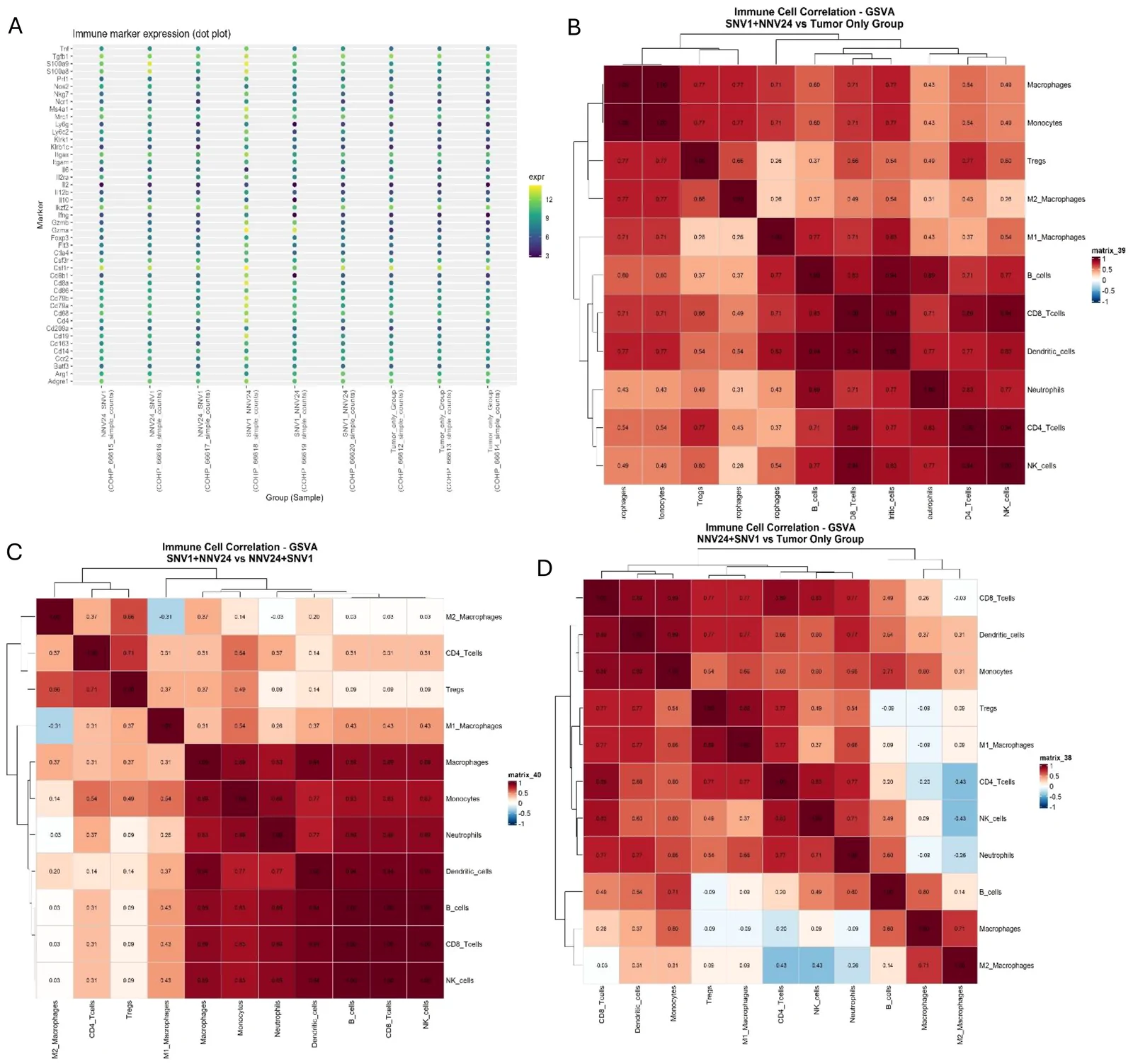
Immune cell landscape and intercellular correlation analysis across treatment groups. **(A)** Dot plot showing the expression of immune marker genes across all nine samples grouped by condition: NNV24 + SNV1, SNV1 + NNV24, and tumor only group. Each dot represents normalized VST expression, with color intensity indicating expression level. Markers represent key immune cell populations including T cells, NK cells, B cells, macrophages, dendritic cells, neutrophils, and monocytes. **(B–D)** Spearman correlation heatmaps of immune cell type enrichment scores (GSVA) for each pairwise comparison. Color scale ranges from blue (negative correlation, −1) to red (positive correlation, +1), with numerical values displayed within each cell. Hierarchical clustering was applied to both rows and columns. **(B)** SNV1+NNV24 vs. tumor only group. **(C)** SNV1+NNV24 vs. NNV24+SNV1. **(D)** NNV24+SNV1 vs. tumor only group.

Immune marker expression analysis revealed broad immune activation in both treatment groups relative to the tumor-only group (**Figure 5A**). However, the NNV24+SNV1 group demonstrated consistently strong and more uniform expression of cytotoxic effector markers, including *Gzma*, *Gzmb*, *Prf1*, and *Ifng*, across all three biological replicates, suggesting more robust CD8+ T cell and NK cell engagement. In contrast, the SNV1+NNV24 group showed more heterogeneous marker expression, with notably weaker induction of these cytotoxic programs. Pro-inflammatory macrophage markers (*Nos2*, *Il12b*, *Il6*) and antigen presentation markers (*Cd86*, *Itgax*, *H2-Aa*) were also more consistently elevated in the NNV24+SNV1 group, indicative of stronger M1 macrophage and DCs activation.

GSVA correlation analysis further distinguished the two treatment sequences at the level of immune cell coordination. In the SNV1+NNV24 vs tumor only comparison (**Figure 5D**), immune populations displayed broadly positive and uniform correlations, reflecting a generalized but poorly differentiated immune activation state lacking functional specialization. Strikingly, the NNV24+SNV1 vs. tumor only comparison (**Figure 5D**) revealed a fundamentally different immune architecture, characterized by strong positive co-regulation between cytotoxic effector populations (CD8+ T cells, NK cells) and myeloid effector populations (M1 macrophages, DCs, monocytes), alongside markedly reduced correlation between these effector populations and immunosuppressive compartments (M2 macrophages, Tregs). This pattern is consistent with a coordinated and functionally polarized anti-tumor immune state.

Direct head-to-head comparison of the two treatment sequences (**Figure 5C**) confirmed that NNV24+SNV1 induced a qualitatively distinct immune configuration, with negative or near-zero correlations observed between M1 macrophages and Tregs, and between cytotoxic effectors and M2 macrophages, indicating active antagonism between pro- and anti-inflammatory immune programs. This immune polarization was absent in the SNV1+NNV24 group. Together, these data suggest that priming with the neoantigen vaccine NNV24 before SNV1 adjuvant administration establishes a cytotoxic and pro-inflammatory immune foundation that is subsequently amplified, whereas the reverse sequence fails to achieve this coordinated polarization. Treatment sequence therefore critically determines not only the magnitude but the functional quality of immune TME remodeling, with NNV24+SNV1 representing the superior immunological strategy.

### 3.5 DC maturation and T cell function

**Figure 6:**
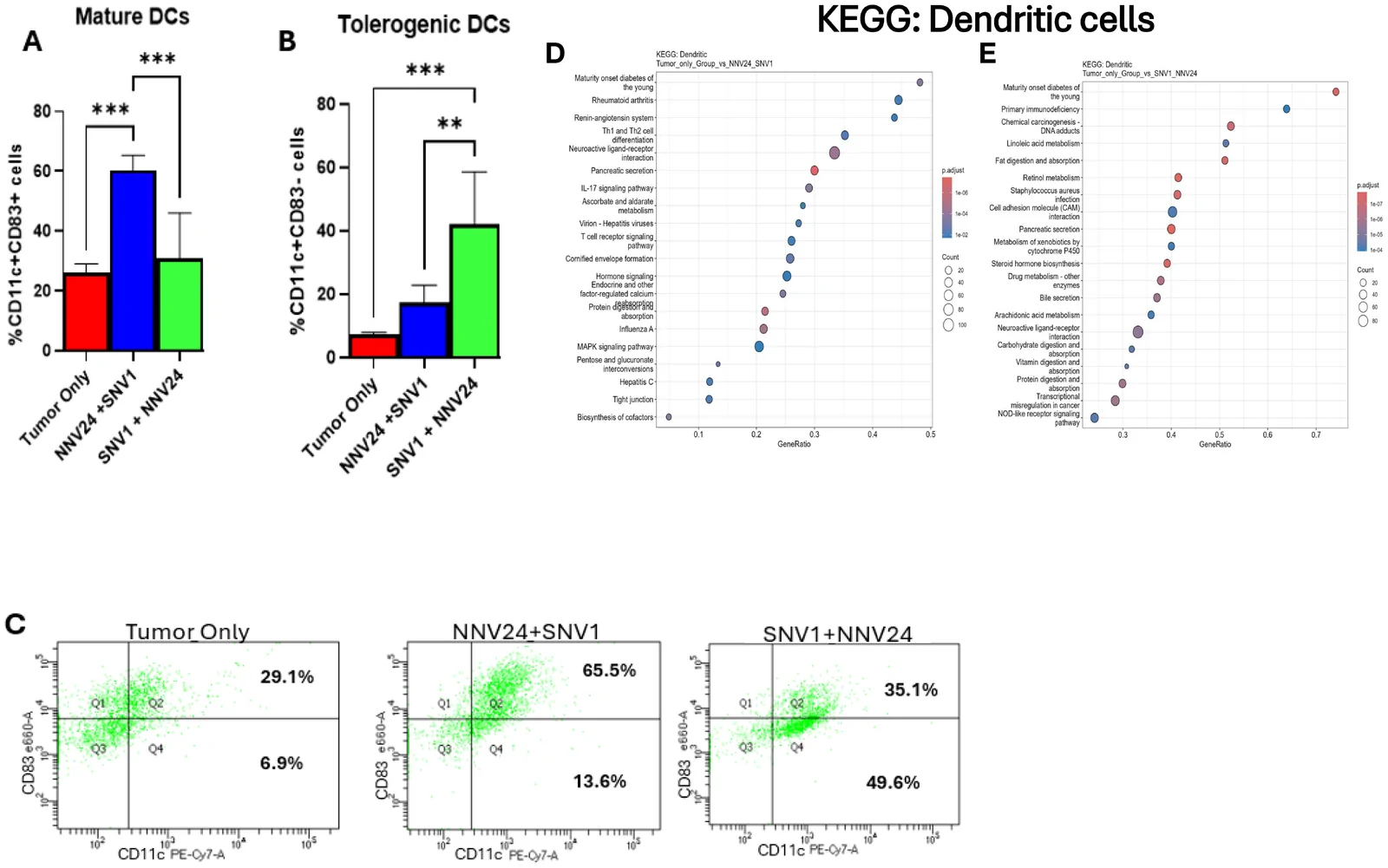
Mature and tolerogenic DCs and KEGG pathway analysis. **(A)** Percentage of mature DCs (CD11c^+^CD83^+^) across treatment groups: Tumor only, NNV24 + SNV1, and SNV1 + NNV24 (***p < 0.0001; ***p < 0.001; **p < 0.01). **(B)** Percentage of tolerogenic DCs (CD11c^+^CD83^-^) across the same treatment groups (***p < 0.001; **p < 0.01; ns = not significant). **(C)** CD11c^+^ CD83^+^ and CD11c^+^ CD83^-^ flow cytometry dot plot showing quadrant gating of major DC subsets. **(D, E)** KEGG-based Gene Set Enrichment Analysis (GSEA) for DC-related pathways across different comparisons: **(D)** Tumor Only vs NNV24+SNV1, **(E)** Tumor Only vs SNV1+NNV24. Dot plot displays significantly enriched pathways associated with immune signaling and metabolic processes. Dot size represents gene count per pathway, and color indicates adjusted p-value (p.adjust).

**Figure 7:**
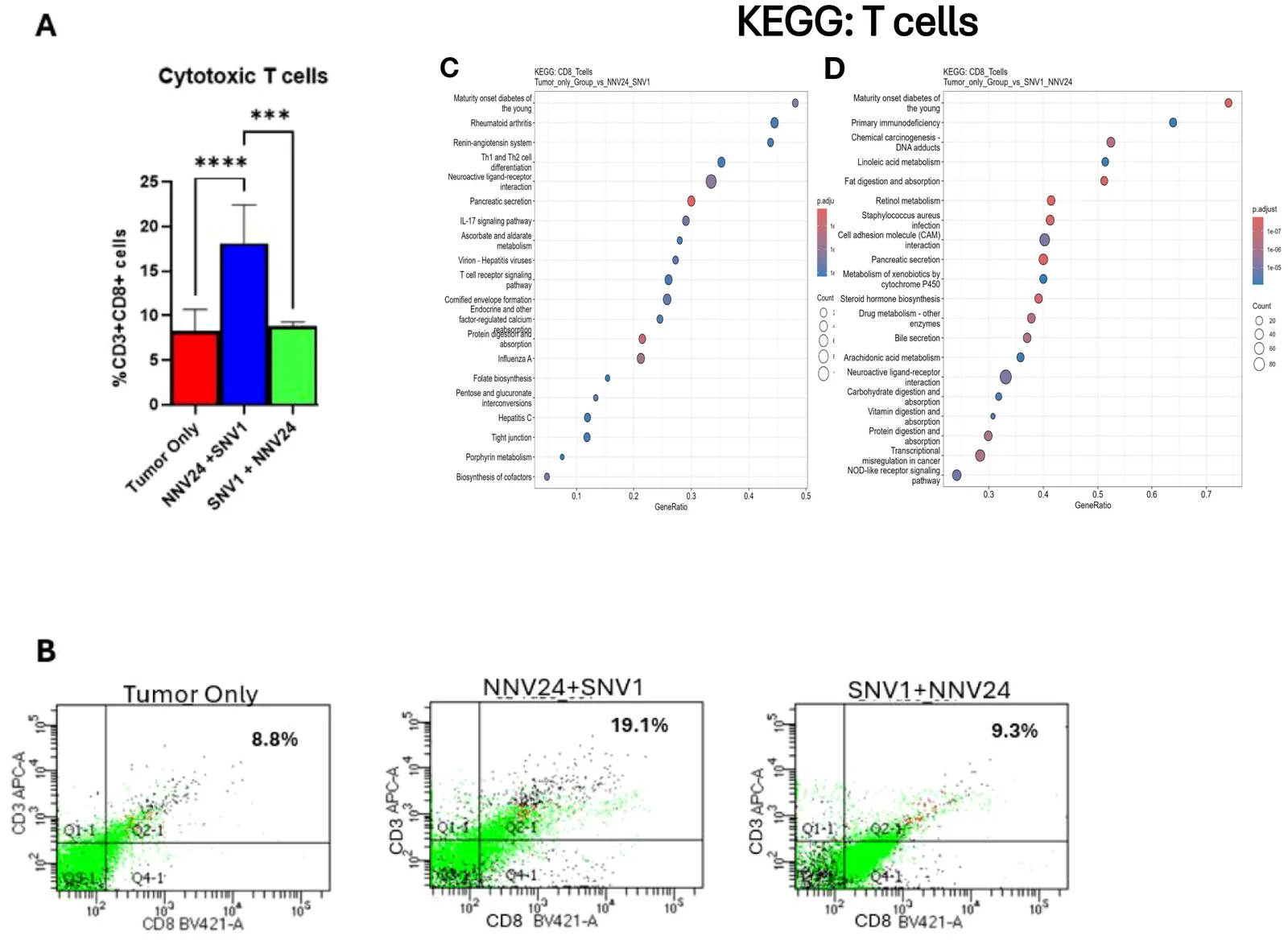
T-cell analysis and KEGG pathway enrichment. **(A)** Percentage of cytotoxic T cells (CD3^+^CD8^+^) across treatment groups: Tumor Only, NNV24 + SNV1, SNV1 + NNV24, and NNV24 + SNV1 (2×). Combination therapies significantly increased CD8^+^ T cell infiltration compared to tumor only controls (***p < 0.001, ****p<0.0001). **(B)** CD3^+^ CD8^+^ flow cytometry dot plot showing quadrant gating of major T cell subsets. **(C, D)** KEGG-based Gene Set Enrichment Analysis (GSEA) for immune-related pathways associated with CD8^+^ T cells across different comparisons: **(C)** Tumor only vs. NNV24+SNV1, and **(D)** tumor only vs. SNV1+NNV24. Dot plots display enriched pathways, with dot size representing gene count and color indicating adjusted p-value (p.adjust). Pathways include T cell receptor signaling, cytokine–cytokine receptor interaction, and immune checkpoint regulation.

The DC compartment provides the clearest mechanistic link between adenovirus-first sequencing and downstream effector immunity. NNV24+SNV1 produced the highest frequency of mature CD11c^+^CD83^+^ DCs and the lowest frequency of tolerogenic CD11c^+^CD83^-^ DCs among all groups (**Figures 6A–C**), and DC-associated transcriptomes were enriched for antigen-processing and cytokine-signaling pathways in the tumor-only versus NNV24+SNV1 comparison (**Figures 6D, E**). Because mature, non-tolerogenic DCs are the principal drivers of CD8^+^ T-cell cross-priming, this DC phenotype is the plausible cellular substrate connecting early adenoviral antigen release to the CD3^+^CD8^+^ effector dominance we observed in the same regimen (**Figure 7**). The reciprocal reduction in tolerogenic DCs is mechanistically important: it indicates that adenovirus-first sequencing does not merely add mature DCs but actively shifts the DC compartment away from a tolerance-promoting state, removing a brake on effector priming that the reverse sequence leaves intact.

Flow cytometry revealed that NNV24+SNV1 achieved the highest proportion of CD3^+^CD8^+^ cytotoxic T cells, surpassing SNV1+NNV24 regimens and controls (**Figures 7A, B**). KEGG analysis of CD8^+^ T-cell transcriptomes confirmed activation of Th1 and Th2 cell differentiation, T cell receptor signaling and IL-17 signaling pathways in NNV24+SNV1 vs. SNV1+NNV24 treated mice (**Figures 7C, D**).

### 3.6 Macrophage reprogramming and apoptosis

**Figure 8:**
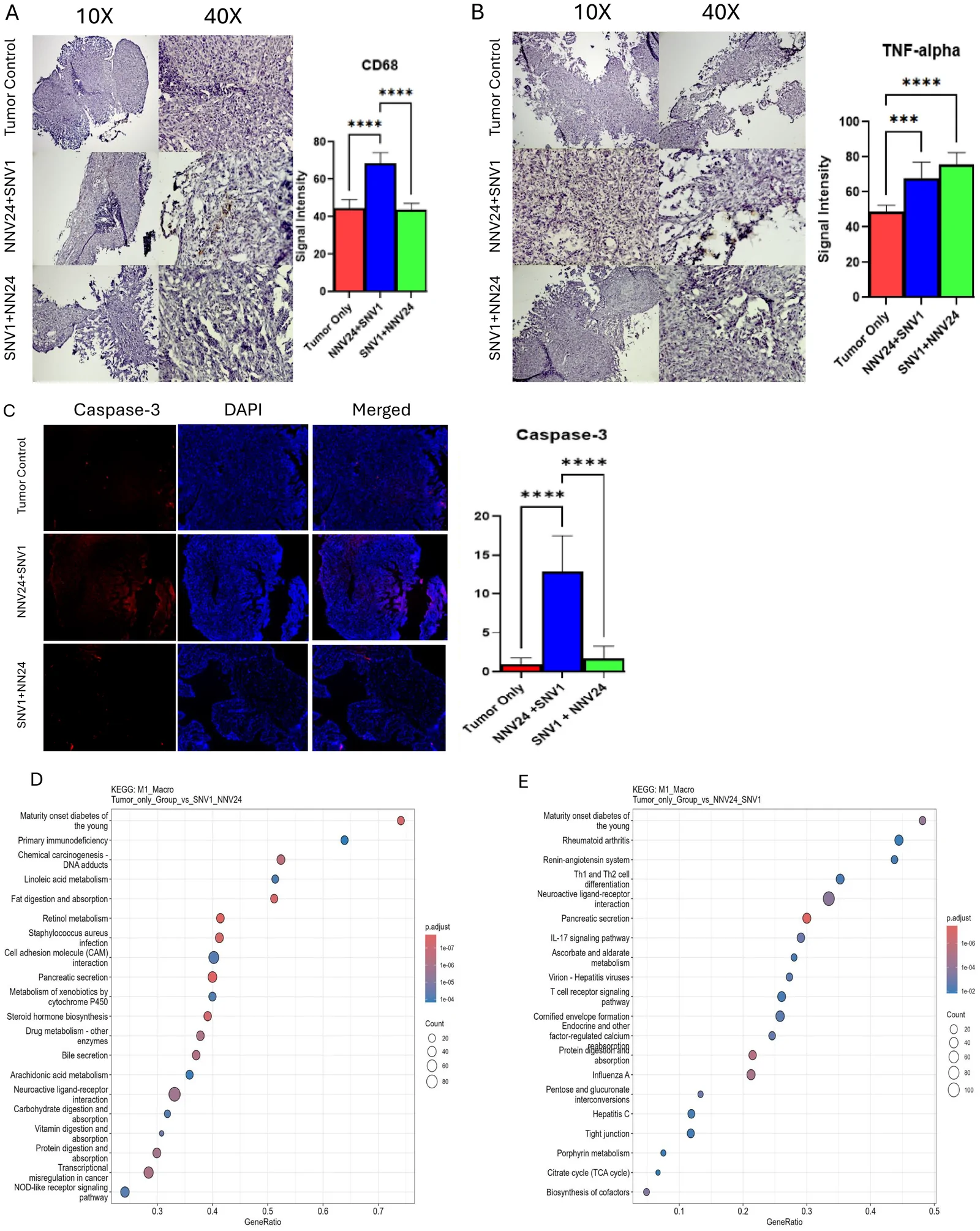
IHC and transcriptomic analysis of TME. **(A)** Immunohistochemical staining for CD68 (macrophage marker) in tumor sections from different treatment groups: Tumor Only, NNV24 + SNV1, and SNV1 + NNV24. Representative images show CD68-positive cells (brown staining) with corresponding quantification of signal intensity. Combination therapies significantly reduced CD68 expression compared to tumor only (*p < 0.05). **(B)** IHC staining for TNF-α in tumor sections across treatment groups. Bar graph indicates signal intensity, with combination viral treatments showing reduced TNF-α expression relative to controls (*p<0.05). **(C)** Immunofluorescence staining for Caspase-3 (red) with nuclear counterstain (DAPI, blue) and merged images. Quantification of signal intensity demonstrates increased Caspase-3 expression in combination therapy groups, indicating enhanced apoptosis (***p < 0.0001, ****p < 0.0001). **(D)** KEGG GSEA for Tumor Only versus SNV1 + NNV24 treatment groups. Enriched pathways include immune regulation, lipid metabolism, and xenobiotic processing, highlighting functional shifts induced by viral therapy. **(E)** KEGG-based Gene Set Enrichment Analysis (GSEA) comparing tumor only vs. NNV24 + SNV1 treatment groups. Dot plot displays significantly enriched pathways associated with immune signaling and metabolic processes. Dot size represents gene count per pathway, and color indicates adjusted p-value (p.adjust).

IHC showed the lowest CD68^+^ macrophage density and the lowest TNF-α signal in the SNV1+NNV24 group (**Figures 8A, B**). In the immunosuppressive ovarian TME, CD68^+^ cells are dominated by tumor-supportive, M2-like tumor-associated macrophages, and TNF-α in this setting is low expressed. The reduced CD68^+^ burden therefore most likely reflects contraction of the suppressive myeloid compartment rather than loss of beneficial inflammation. Thus, the lower CD68^+^/TNF-α signal in SNV1+NNV24 suggests lower M1 macrophage compared to NNV24+SNV1.

Caspase-3 immunofluorescence increased in a stepwise manner, peaking in NNV24+SNV1 (**Figure 8C**). Because caspase-3 integrates both direct oncolysis and immune-mediated killing, its co-occurrence with peak CD8^+^ infiltration and mature-DC frequency in the same regimen is consistent with a model in which adenoviral priming maximizes early immunogenic cell death, which in turn feeds antigen to the licensed DC compartment ahead of the vaccinia boost.

Immune deconvolution confirmed enrichment of cytotoxic subsets with concomitant depletion of macrophage signatures (**Figure 8D**), and KEGG GSEA of macrophage transcriptomes showed downregulation of TNF, NF-κB, and cytokine–cytokine receptor signaling alongside enrichment of oxidative phosphorylation and fatty acid metabolism in NNV24+SNV1 relative to SNV1+NNV24 (**Figures 8D, E**). SNV1+NNV24 showed enhanced immune deficiency pathway while NNV24+SNV1 group showed enhanced immunostimulatory related pathways which support our IHC findings.

## 4 Discussion

### 4.1 Key finding and conceptual advance

This study demonstrates that the order of heterologous OV administration is a decisive variable for therapeutic outcome in OC. Using clinically relevant stem cell–delivered adeno and vaccinia OV platforms we show that adeno virus (NNV24) first, vaccinia (SNV1) second sequencing markedly improves survival, increases viral localization within tumors, and amplifies antitumor immunity compared with the reverse order. These data illuminate *how* sequencing sculpts oncolysis, antigen handling, myeloid programming, and cytotoxic effector dominance to produce synergy rather than simple additivity (11, 23, 27, 28).

### 4.2 Mechanistic basis for sequence dependent efficacy

#### 4.2.1 Adenovirus priming enhances intratumoral infection and immunogenic cell death

CRAd-S-pk7 uses the survivin promoter to enforce -to enforce tumor selective replication and a pk7 polylysine fiber to target heparin sulfate proteoglycans on cancer cells, enabling efficient infection and early tumor debulking with release of tumor antigens and danger signals (6, 29, 30). NSCs further shield viruses from neutralization and improve tumor tropism and intratumoral hand-off. Together, these features provide a strong mechanistic basis for effective priming when adenovirus is given first (11, 31).

#### 4.2.2 Histopathologic findings reflect enhanced oncolytic engagement and ICD

The broad viral distribution we observed aligns with prior work showing NSC delivery increases OV persistence and spread *in situ* (31). Suppressed Ki-67, increased cleaved caspase3, and tissue disruption/necrosis are canonical readouts of OV cytotoxicity and ICD, which liberates ATP/HMGB1, exposes calreticulin, and conditions DCs to mature and cross prime CD8^+^ T cells (32, 33).

#### 4.2.3 Adenovirus directly licenses DCs

Adenoviral infection and adenovirus-conditioned tumor exosomes drive DC maturation and IL-12-dependent T- and NK-cell activation, providing a mechanistic link between adenoviral priming and efficient antigen presentation before the boost (34, 35). This licensing step is directly reflected in our own data. Mice receiving NNV24 (the adenovirus-based platform) first showed the highest frequency of mature CD11c^+^CD83^+^ DCs and the lowest frequency of tolerogenic CD11c^+^CD83^-^ DCs among all groups (**Figures 6A–C**), and KEGG GSEA of the DC compartment in the adenovirus-first comparison (Tumor Only vs NNV24+SNV1; **Figure 6D**) showed enrichment of DC-associated immune pathways relative to control. The adenovirus-licensed, non-tolerogenic DC population established during priming is therefore the plausible cellular substrate on which the subsequent vaccinia boost acts to expand the CD3^+^CD8^+^ effector response observed in the same regimen (**Figure 7**). This directly ties the subsection to the central finding of the study: the superiority of adenovirus-first sequencing is mechanistically explained, at least in part, by its capacity to license DCs for effective cross-priming before the immunodominant vaccinia boost is delivered (34, 35).

On the other hand, Oncolytic vaccinia virus administered prior to oncolytic adenovirus may induce a robust type I interferon-driven antiviral state and activate NK-cell-mediated antiviral immunity, thereby limiting subsequent adenoviral infection and replication (36, 37).

### 4.3 Vaccinia as an amplification boost

Our IHC data show that administering either virus first reduced intratumoral localization of the second (**Figures 3B, C**), providing direct evidence of interference between the two lytic cycles. The direction of this interference is asymmetric and explains why order matters. When adenovirus is given first, its slower, tumor-restricted, survivin-driven replication debulks the tumor, releases antigen, and licenses DCs without generating the intense antiviral state that would suppress an incoming virus; the subsequent vaccinia cycle therefore proceeds in an already-inflamed but not yet antivirally refractory microenvironment and functions as an effective boost. When vaccinia is given first, its rapid, highly immunogenic replication elicits strong innate antiviral signaling and accelerated immune contraction (**Figure 4A**), which restricts productive adenoviral replication and antigen presentation during the second cycle. Ratto-Kim, Currier (38) Showed that adenovirus prime followed by Modified Vaccinia Ankara boost induced stronger HIV-specific CD8 T-cell responses than homologous regimens. This finding supports our findings.

Vaccinia is strongly immunogenic and, when delivered by MSCs, is protected from complement and neutralizing antibodies, enabling robust intratumoral amplification and spread that “boosts” the primed response. Preclinical and translational data with vaccinia show improved viral persistence, abscopal effects, and increased tumor infiltrating lymphocytes when vaccinia is shielded by a carrier cell (23, 39).

### 4.4 Immune remodeling and effector dominance

#### 4.4.1 Effector cell enrichment and pathway activation

Our GSVA/flow results, including an increased CD8^+^ T cells and NK cells, with reduced M2 macrophages and tolerogenic DCs, are consistent with OV literature showing that (I) adenoviral priming promotes antigen processing/presentation and TCR signaling and (II) vaccinia amplification deepens intratumoral inflammation and cytotoxic infiltration (34, 40, 41).

#### 4.4.2 Macrophage reprogramming and microenvironment normalization

Adenovirus first reshapes the myeloid landscape by shifting -tumor associated macrophages (TAMs) away from an immunosuppressive, tumor supportive, phenotype toward a more immunostimulatory state (42). The increase in CD68 and TNFα staining, together with reduced macrophage signatures from deconvolution analysis, indicates a broad contraction of the myeloid suppressive compartment. This is consistent with evidence that OVs can disrupt the metabolic programs that stabilize M2-like TAM identity and instead promote conditions that favor antigen presentation, inflammatory signaling, and improved T-cell infiltration (43). By alleviating these myeloid- derived inhibitory cues and reducing the metabolic rigidity associated with M2 polarization, the adenovirus first regimen produces a more permissive, immune reactive TME that supports durable effector cell persistence and antitumor activity (44–46).

#### 4.4.3 Context within OV sequencing literature

Although formal studies specifically testing adenovirus and vaccinia sequencing are limited, extensive work in heterologous prime boost immunology shows that initiating treatment with a vector optimized- for antigen release and DC engagement, then boosting with a highly immunogenic virus, consistently maximizes CD8^+^ T-cell responses and therapeutic potency (47). This principle is reinforced across OV engineering and vaccine platforms, where frontloading antigen availability and innate licensing improves the performance of subsequent viral boosts. These broader findings align closely with our mechanistic model and provide a conceptual framework supporting the superiority of adenovirus- first- sequencing in this study.

#### 4.4.4 Limitations and future directions

Several limitations of this study should be acknowledged and define priorities for future work. First, our conclusions derive from the syngeneic, immunocompetent ID8 model. Although ID8 recapitulates the intraperitoneal dissemination, omental tropism, ascites formation, and macrophage-dominated immunosuppressive niche that characterize advanced human ovarian cancer, it is a single model of murine origin. The generalizability of the adenovirus-first sequencing benefit to other ovarian cancer contexts, particularly human high-grade serous lines that model advanced disease and malignant ascites, such as SKOV-3 and OVCAR-3/OVCAR-8 will require validation, ideally in immunocompetent or humanized settings that preserve the immune interactions central to the mechanism we describe. Because SKOV-3 and OVCAR lines are human and require immunodeficient hosts, dedicated syngeneic or humanized models will be needed to test whether the immune-dependent component of the sequence effect is retained.

Second, while our design isolates the effect of sequence, direct monotherapy arms (adenovirus alone, vaccinia alone) at matched MOI would further delineate the relative contribution of each platform to the combination benefit; the MOI used here (50 for CRAd-S-pk7, 1 for VP001) reflects validated carrier-loading conditions but was not systematically titrated *in vivo*.

Third, the safety profile of combined SNV1 and NNV24 treatment was not systematically evaluated. While no overt toxicity was observed during the experiments, formal assessments of off-target viral infection, cytokine-mediated toxicities, viral biodistribution, and potential effects on normal tissues were not performed. Furthermore, interactions between two replicating oncolytic viruses may produce biological effects that differ from those observed with either agent alone. Therefore, future studies are warranted to comprehensively assess toxicity, pharmacodynamics, biodistribution, and immunological safety before clinical translation of this combination approach.

Finally, although KEGG and Reactome pathway analyses were valuable for hypothesis generation regarding metabolic and immune reprogramming, they are correlative; targeted functional validation (e.g., macrophage metabolic assays, DC adoptive transfer, or CD8 depletion) will be required to establish causality. Addressing these points in additional tumor models with formal toxicology will strengthen the translational case for adenovirus-first heterologous OV sequencing.

## 5 Conclusion

This study demonstrates that the sequence of heterologous OV administration significantly impacts therapeutic outcomes in OC. Initiating treatment with NSC-delivered adenovirus (NNV24) followed by MSC-delivered vaccinia virus (SNV1) significantly improves survival, enhances viral localization, and promotes robust immune activation, including increased cytotoxic T-cell infiltration and DC maturation, while reducing tolerogenic subsets. In contrast, reversing the sequence attenuates these effects, likely due to viral interference. These findings provide a mechanistic rationale for sequencing strategies in next generation OV based immunotherapy and support further clinical evaluation of adenovirus-first regimens for optimal antitumor efficacy.

## Data Availability

The raw data supporting the conclusions of this article will be made available by the authors, without undue reservation.
